# Effects of home bleaching agents on hybrid ceramics: mechanical properties and color change

**DOI:** 10.1186/s12903-024-05336-w

**Published:** 2025-01-08

**Authors:** Waleed M. Hafny, Imam M. Ibrahim, Mohamed El-Demellawy, Hoda M. Abdel Sadek

**Affiliations:** Department of Fixed Prosthodontics – Faculty of Dentistry, Ain Shams University, Organization of African Unity, St, El-Qobba Bridge, El Weili, Cairo Governorate, Egypt

**Keywords:** Biaxial flexural strength, Color change, Home bleaching, Hybrid ceramics, Restorative dentistry

## Abstract

**Background:**

Home bleaching is a promising option for addressing discolored teeth conservatively. However, its impact on the physical and mechanical properties of indirect restorations remains unknown. This study provides comparative insights into the material responses to aesthetic treatments by assessing the effects of home bleaching agents on two hybrid ceramics: VITA ENAMIC^®^ and Grandio Blocs. The focus is on evaluating color stability and biaxial flexural strength.

**Methods:**

60 discs (12 × 1 mm) were prepared from hybrid ceramics. Specimens were divided into two groups according to type of hybrid ceramic (30 specimens in each group); group A: VITA ENAMIC^®^, group B: Grandio Blocs. Each group was further subdivided into 3 subgroups according to the concentration of home bleaching agents used (10 specimens in each subgroup); subgroup C: unbleached control group, subgroup C10%: carbamide peroxide 10% bleaching agent, and subgroup C35%: carbamide peroxide 35% bleaching agent. Samples of subgroup C10% were bleached with Home Bleaching Opalescence PF (10%) for 8 h per day for 14 days, as directed by the manufacturer. Samples of subgroup C35% were bleached using WHITE*smile* Carbamide Peroxide (35%), following the manufacturer’s directions for 30 min every day for 14 days. Color change (ΔE) was assessed using of the CIE L*a*b* system. Then, the biaxial flexural strength test was done. The data were analyzed using two-way ANOVA tests and Weibull analysis.

**Results:**

VITA ENAMIC samples exhibited significantly higher color change than Grandio Blocs (*p* < 0.001), while Grandio Blocs showed significantly higher biaxial flexural strength (*p* < 0.001). The concentration of the bleaching agent had no significant effect on color change (*p* = 0.086). However, regardless of its concentration, bleaching significantly reduced biaxial flexural strength in both materials (*p* < 0.001). Weibull analysis showed that Grandio Blocs had higher characteristic strength, while VITA ENAMIC demonstrated more reliable failure behavior.

**Conclusions:**

Compared to Grandio Blocs, VITA ENAMIC^®^ exhibited a greater color change with both 10% and 35% carbamide peroxide home bleaching agents. Additionally, both concentrations of carbamide peroxide reduced the biaxial flexural strength of hybrid ceramics. These findings can guide clinicians in selecting hybrid ceramics based on clinical demands for aesthetics and durability. VITA ENAMIC^®^ offers greater structural reliability and predictability for aesthetic restorations, while Grandio Blocs provide superior durability and stress resistance for high-stress clinical applications.

## Background

Aesthetic CAD/CAM materials have advanced significantly, transforming treatments from two-step, bi-layered, high-strength ceramic restorations to single-step, monolithic restorations that eliminate the challenges of veneering chipping and fractures [1]. Patients’ demand for nonmetallic materials has increased dramatically in recent years, often due to metal anxiety disorders or reported allergies [2]. Ceramics, composite resins, and polymers are examples of metal-free materials that are gaining popularity. Ceramics are commonly chosen for their inert properties and aesthetic appeal, being biocompatible, strong, and visually pleasing, with high fracture resistance and low material wear [3]. However, they are brittle and abrasive. In contrast, polymers have a low modulus of elasticity, allowing them to absorb stress through deformation. To combine the benefits of ceramics and polymers, ceramic-polymer composites were developed, offering improved quality for CAD/CAM systems [4]. These composites retain the color stability and durability of ceramics, while benefiting from the enhanced flexural strength and low abrasiveness of resin composites. Furthermore, they are repairable and chemically compatible with adhesive resin cement [5].

Polymer-infiltrated ceramic network (PICN) is created by immersing pre-sintered porous ceramics in a resin monomer, which is then polymerized. The analysis of PICN reveals a dominant ceramic network, primarily composed of leucite, with zirconia as a minor phase, all embedded in a polymer-based network [6]. This dual-network microstructure results in properties that lie between those of pure resins and ceramics, with flexural strength comparable to dentin but lower compressive strength [7]. Nano-ceramic hybrid materials, which combine ceramic nanosized particles with larger filler particles, enhance strength by preventing crack propagation. Materials such as resin nanoceramic blocks (RNC) offer fracture toughness and aesthetic qualities similar to those of traditional composite resin materials [8, 9].

Bleaching is a low-risk, conservative, and aesthetically pleasing method for eliminating tooth discoloration. The process can be performed either at home or in a dental office. In at-home bleaching, hydrogen peroxide or carbamide peroxide are the active ingredients in many of the products available on the market. The oxidation process generated by these bleaching agents may negatively affect dental restorations [10]. One of the main disadvantages of CAD/CAM hybrid ceramics is their susceptibility to color change. However, they offer better color stability than resin composites, thanks to their high degree of polymerization and improved mechanical properties [11].

Biomaterials are widely used in dental composite materials, and their physical, mechanical, wear, and biocompatibility properties must be thoroughly evaluated before clinical application. Significant research has focused on developing various approaches to modify the microstructure of dental biomaterials [12]. For indirect restorations, long-term aesthetic success depends on factors such as color stability and durability. Color change is a critical consideration, as it directly impacts the appearance of restorations over time [13]. Additionally, flexural strength plays a crucial role in the longevity of these materials, as higher flexural strength allows restorations to withstand greater forces [14]. Home bleaching agents, commonly used for aesthetic purposes, may alter the surface morphology and mechanical properties of existing indirect restorations. However, there is limited comparative data on the effects of bleaching on hybrid ceramic materials. Therefore, the aim of this study is to evaluate the impact of home bleaching agents on the color change and biaxial flexural strength of hybrid ceramics, such as VITA ENAMIC^®^ and Grandio Blocs. This study seeks to enhance our understanding of how bleaching treatments influence the performance of hybrid ceramics in clinical settings. The objectives are to investigate the effects of home bleaching systems on color change and biaxial flexural strength in hybrid ceramic materials. The null hypotheses tested were that home bleaching systems would not affect the color change or biaxial flexural strength of these materials.

## Materials

The materials used in this study, lot numbers, manufacturer and compositions are listed in Table 1.

**Table 1:** The materials used in this study, lot numbers, manufacturer, and compositions

Material	Composition	Manufacturer	LOT Number
VITA ENAMIC	Polymer infiltrated (Urethane dimethacrylate, Triethylenglycoldimethacrylat 14 wt%) feldspar ceramic network (86 wt%)	VITA Zahnfabrik Postfach 1338 D-79,704 Bad Sackingen, Germany	95,080
Grandio Blocs	Resin nano ceramic. (86 wt% inorganic fillers; particle size 20–60 nm), embedded in a polymer matrix (14% UDMA + DMA)	VOCO GmbH, Anton-Flettner-Straße 1-3, 27,472 Cuxhaven, Germany	2,313,294
Opalescence Home bleaching	10% carbamide peroxide	Opalescence teeth whitening 505west ultradent Drive south jordan ut84095	BSCPB
WHITE*smile* Home bleaching	35% carbamide Peroxide	WHITE-smile GmbH Weisenheimer Strafe 6 69,488 Birkenau Deutschland	23,028

### Methods

#### Sample grouping

A power analysis was designed to have adequate power to apply a statistical test of the null hypothesis that there is no difference between tested groups regarding biaxial flexural strength. By adopting an alpha (α) level of 0.05 (5%), a beta (β) level of 0.2 (i.e., power = 80%), and an effect size (f) of 0.552 calculated based on the results of a previous study [15], the predicted total sample size (n) was found to be 60 samples (i.e., 30 samples per group and 10 samples per subgroup). The sample size calculation was performed using G*Power version 3.1.9.7 [16].

60 discs (12 × 1 mm) were prepared from hybrid ceramics. Specimens were divided into two groups according to type of hybrid ceramic (30 specimens in each group); group A: VITA ENAMIC^®^, group B: Grandio Blocs. Each group was further subdivided into 3 subgroups according to the concentration of home bleaching agents used (10 specimens in each subgroup); subgroup C: unbleached control group, subgroup C10%: carbamide peroxide 10% bleaching agent, and subgroup C35%: carbamide peroxide 35% bleaching agent.

### Sample fabrication

**Fig. 1:**
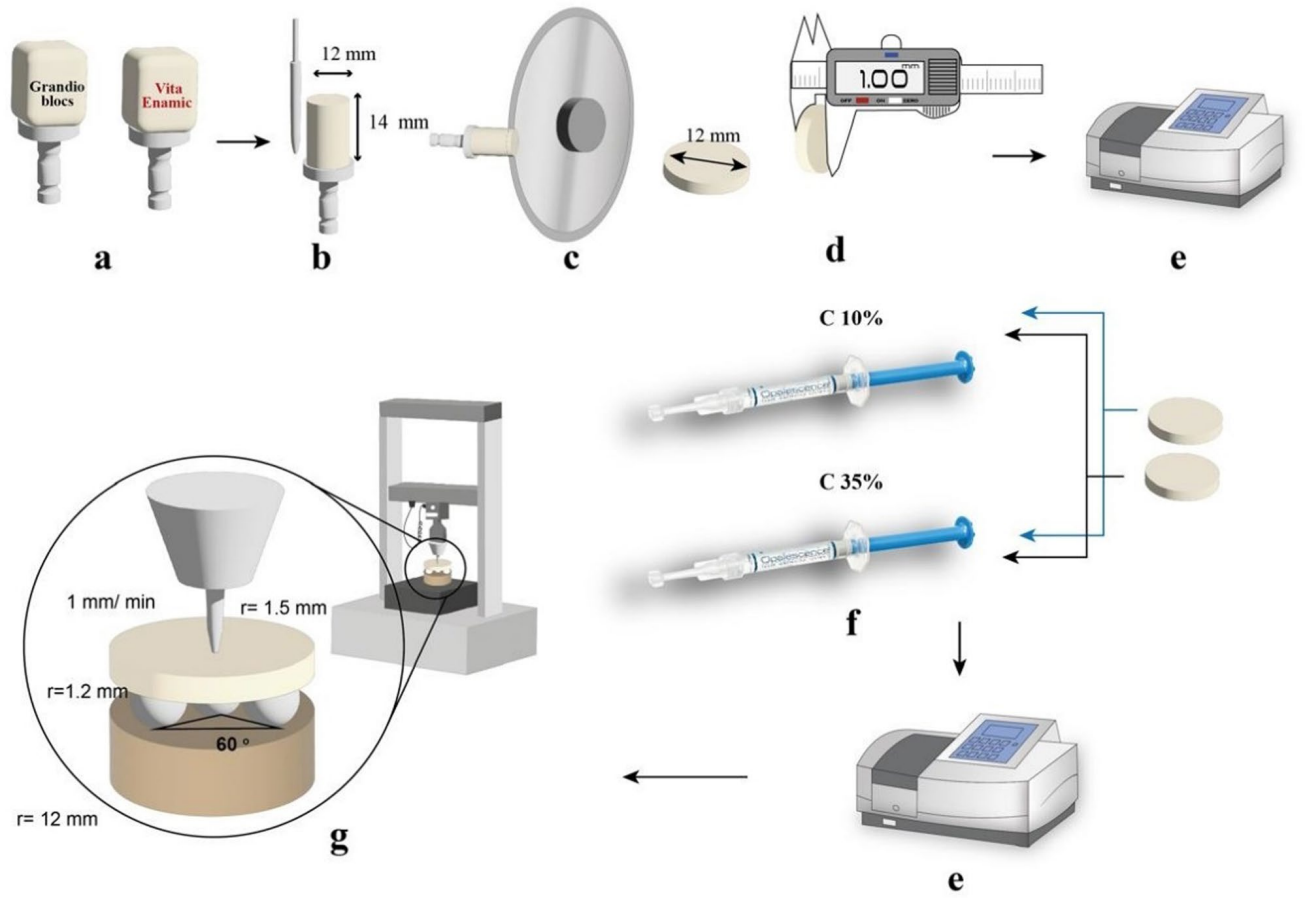
Flowchart for the specimen preparation, Bleaching technique, and testing protocol; **a**: hybrid ceramic blocks, **b**: roundation of blocks, **c**: IsoMetTM 4000 linear precession saw, **d**: checking sample size with Insize Digital Caliper, **e**: spectrophotometer, **f**: Bleaching agent application, **g**: Biaxial flexural strength using universal testing machine

VITA ENAMIC^®^ (VITA Zahnfabrik, Germany) and Grandio Blocs (GR; VOCO, Cuxhaven, Germany) were in the form of blocks (Fig. 1a). The shades selected for the blocks were A2 for the Grandio Blocs and 2M2 for VITA ENAMIC^®^, as they are the most frequently selected shades representing the natural tooth color [17]. Three blocks of each material were first ground into cylinders with a diameter of 12 mm using the Universal Tool Grinder Machine (C40, Sung Kwang Machinary-Siheung, Korea) (Fig. 1b). The cylinders of each material were then sliced into discs of thickness 1 mm (Fig. 1c). The slicing was done using an IsoMetTM 4000 linear precession saw (Lake Bluff, Illinois, USA) with a diamond disc (IsoMetTM Buehler, thickness 0.3 mm, diameter 127 mm) using a speed of 2500 rpm and a feed rate of 13.7 mm/min. The polishing of the discs was done using the VITA ENAMIC^®^ polishing set (VITA Zahnfabrik, Germany). The pink-coded pre-polishing tool was used, and then the grey-coded high-gloss polishing tool was used. Polishing was done in one direction to achieve a glossy surface [18]. Grandio Blocs samples were polished using the Standard Composite Finishing and Polishing Kit (Dimanto, Voco GmbH, Germany) [19]. The specimen dimensions were confirmed to an accuracy of ± 0.1 mm using a digital caliper (Fig. 1d).

### Bleaching technique

The specimens of each material were randomly assigned to two subgroups using simple randomization, with each subgroup receiving a different home bleaching agent. For subgroup C10%, home bleaching Opalescence PF Carbamide peroxide (10%) was used according to the manufacturer’s instructions for 8 h per day for 14 days (Fig. 1f) [20]. While for subgroup C35%, home bleaching WHITESmile Carbamide Peroxide (35%) was used according to the manufacturer’s instructions for 30 min per day for 14 days (Fig. 1f) [21]. After each application of both bleaching agents, the discs were rinsed with water for 20 s until the gel was removed, then air-dried and left untouched until the next application.

### Color change measurement

The Vis-NIR spectrophotometer (Agilent Cary 5000 spectrophotometer, Agilent, USA) was used to measure color for each group before and after bleaching agent application, according to a quality management system certified to ISO 9001 (Fig. 1e) [22]. The examiner conducting the spectrophotometric measurements was blinded to the group assignments. The spectrophotometer measures the ratio of light reflected from a sample to that reflected from a reference white across the visible spectrum at intervals of 1, 5, 10, or 20 nm. The results are expressed by the spectral reflectance function. The wavelength scan for these measurements was carried out from 380 nm to 780 nm [23]. Measurements were made according to the traditional CIELab color formula created by the Commission Internationale de l’Eclairage (CIE). L*, a*, and b* stand for lightness, hue, and chroma, respectively. Lightness is represented by L*, which has a value between 0 and 100, where 100 is white and 0 is black. The red-green axis saturation is denoted by a*, and the yellow-blue axis saturation is denoted by b* [24].

The degree of color difference between the compared colors is expressed in ΔE units. The total color difference, according to L*, a*, b* coordinates, is calculated as shown in the equation: (ΔE) was calculated: ΔE* = [(L*1 − L*0) 2 + (a*1 − a*0) 2 + (b*1 − b*0) 2]^1/2^ [25].

### Biaxial flexural strength testing

A piston-on-three-ball (P3B) attachment was used for this test. A cylindrical round end loading pin with a diameter of 1.5 mm was used. A mounting jig was designed to facilitate specimen positioning while maintaining the same relation between the supports and the applied load for all specimens. Each disc specimen was placed centrally on three hardened steel balls of 1.2 mm diameter arranged in an equilateral triangle with a 60º angle which situated inside a support circle with a diameter of 12 mm. The discs were placed concentrically on these steel balls [26]. A universal testing machine (Instron 3365, Instron Corporation) was used to apply the load at a crosshead speed of 1 mm/min until the disc fractured (Fig. 1g). The biaxial flexural strength *σ* (MPa) was then calculated using the following equation [26]: *σ* = −0.2387*P*(*X* − *Y*)/*d*2, where, *d* is the thickness of the specimens (1 mm), *X* = (1 + *ν*)ln(*r*2/*r*3)2 + [(1 − *ν*)/2](*r*2/*r*3), *Y* = (1+*ν*)[1 + ln(*r*1/*r*3)2] + (1 − *ν*)(*r*1/*r*3), where *ν* is Poisson’s ratio for hybrid ceramic materials; *r*1 is the radius of supporting ring in mm ; *r*2 is the radius of the loaded area in mm (0.8 mm); and *r*3 is the radius of the specimen in mm.

### Statistical analysis

Numerical data were presented as mean with 95% confidence intervals (CI), standard deviation (SD), minimum (min.) and maximum (max.) values. Data were tested for normality and variance homogeneity by viewing distribution and using Shapiro-Wilk’s and Levene’s tests, respectively. They were found to be normally distributed and were analyzed using two-way ANOVA followed by Tukey’s post hoc test. Weibull distribution parameters were estimated using the maximum likelihood method [27], and their respective confidence intervals were estimated using Wald’s method [28]. The goodness-of-fit for the Weibull distribution was assessed using the Anderson-Darling test and visually confirmed with Weibull probability plots. The distribution parameters were compared using the chi-square test. The significance level was set at *p* < 0.05 within all tests. Statistical analysis was performed with R statistical analysis software version 4.4.1 for Windows [29].

## Results

Descriptive statistics for the measured variables are presented in Table 2, including mean values, 95% confidence intervals, standard deviations, and ranges for color change and biaxial flexural strength across materials and bleaching treatments. Results of the two-way ANOVA for the two measured outcomes, presented in Table 3, showed no significant interaction effects between the type of hybrid ceramic material and the bleaching agent for either outcome, indicating that the effects of material type and bleaching agent were independent. Therefore, all reported values for the main effects of material type and bleaching agent represent the effects of each variable independently, averaged over the levels of the other variable.

Results showed that only the type of hybrid ceramic material significantly affected color change (*p* < 0.001; Table 3). Main effects comparisons showed that the color change measured in VITA ENAMIC samples (3.10 ± 0.21) was significantly higher (whiter) than that of Grandio Blocs (2.43 ± 0.15) (*p* < 0.001).

For biaxial flexural strength, both the type of hybrid ceramic material and the bleaching agent had significant effects (*p* < 0.001; Table 3). The Grandio Blocs demonstrated significantly higher biaxial strength (342.52 ± 23.36) (MPa) than VITA ENAMIC (172.83 ± 9.84) (MPa). Additionally, unbleached samples (269.48 ± 93.18) (MPa) had significantly higher strength than samples bleached using 10% CP (256.06 ± 88.49) (MPa) and 35% CP (247.49 ± 83.30) (MPa).

**Table 2:** Descriptive statistics

Measurement	Material	Bleaching	Mean	95% CI		SD	Min.	Max.
				Lower	Upper			
Color change from control samples (ΔE)	**Grandio Blocs**	**10% CP**	2.40	2.30	2.50	0.17	2.15	2.63
		**35% CP**	2.46	2.38	2.54	0.13	2.30	2.64
	**VITA ENAMIC**	**10% CP**	3.03	2.90	3.16	0.21	2.52	3.25
		**35% CP**	3.17	3.05	3.29	0.20	2.88	3.44
Biaxial flexural strength (MPa)	**Grandio Blocs**	**Unbleached**	358.87	344.84	372.90	22.63	326.04	390.52
		**10% CP**	341.10	329.08	353.11	19.39	315.99	379.12
		**35% CP**	327.58	316.45	338.71	17.96	295.97	360.05
	**VITA ENAMIC**	**Unbleached**	180.08	175.25	184.91	7.79	168.12	191.30
		**10% CP**	171.02	165.31	176.73	9.21	156.47	185.61
		**35% CP**	167.40	162.15	172.65	8.47	156.04	180.23

CI Confidence Interval, SD Standard deviation, Min. Minimum, Max. Maximum

**Table 3:** Two-way ANOVA test results

Measurement	Parameter	Sum of squares	df	Mean square	f-value	*p*-value
Color change from control samples (ΔE)	**Material**	4.45	1	4.45	140.60	< 0.001*
	**Bleaching**	0.10	1	0.10	3.12	0.086
	Material* bleaching	0.01	1	0.01	0.47	0.497
	**Error**	1.14	36	0.03		
Biaxial flexural strength (MPa)	**Material**	431875.92	1	431875.92	1814.63	< 0.001*
	**Bleaching**	4911.87	2	2455.93	10.32	< 0.001*
	Material* bleaching	867.14	2	433.57	1.82	0.172
	**Error**	12851.83	54	238.00		

df degree of freedom, * significant (*p* < 0.05)

### Weibull distribution

**Fig. 2:**
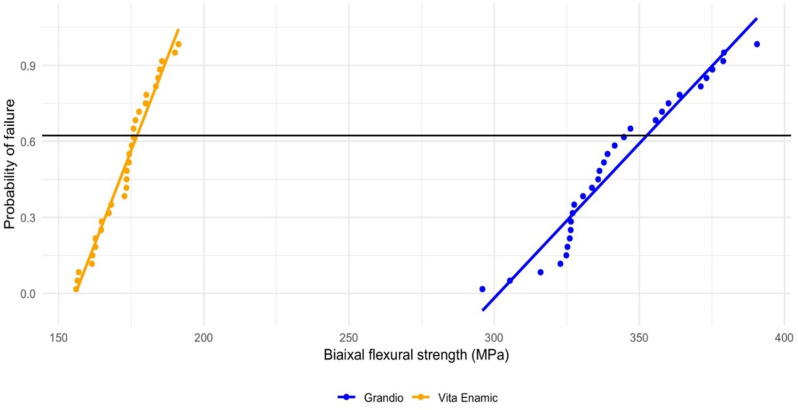
Weibull Probability of failure plot of biaxial flexural strength data

Weibull statistics based on the biaxial flexural strength data are presented in Table 4, and the probability of failure plot is presented in Fig. 2. Results showed a significant difference in the distribution of both materials (*p* < 0.001). Specifically, Grandio Blocs exhibited a Weibull modulus of 15.48 (95% CI: 11.34–19.62), while VITA ENAMIC demonstrated a higher Weibull modulus of 19.55 (95% CI: 14.24–24.86), suggesting that VITA ENAMIC has a more consistent and predictable failure pattern. Additionally, the characteristic strength of Grandio Blocs 353.51 MPa (95% CI: 344.84–362.19) was higher than that of VITA ENAMIC 177.41 MPa (95% CI: 173.97–180.84). This implies that Grandio Blocs has higher overall strength and is less likely to fail under applied stress than VITA ENAMIC.

**Table 4:** Weibull distribution parameters for biaxial flexural strength (MPa)

Parameter	Grandio Blocs	VITA ENAMIC	χ2	*p*-value
Weibull modulus (95%CI)	15.48 (11.34:19.62)	19.55 (14.24:24.86)	202.48	*P* < 0.001*
Characteristic strength (95%CI)	353.51 (344.84:362.19)	177.41 (173.97:180.84)		

CI Confidence Interval, * Significant (*p* < 0.05)

## Discussion

Patients’ aesthetic expectations have significantly increased, leading to a higher demand for dental restorative materials that closely resemble natural teeth [30]. The introduction of new types of dental ceramics, some of which feature resin modifications, has contributed to their increased use in aesthetic dental restorations [31]. Bleaching has long been considered an effective solution for tooth discoloration [32], and the use of bleaching agents has become a common procedure among dentists. However, this widespread use has raised concerns about the potential for undesirable effects and the deterioration of indirect restorations [33]. Bleaching agents can alter the color and mechanical properties of restorative materials, including their flexural strength [34].

The aim of this study was to evaluate the impact of home bleaching agents on the color change and biaxial flexural strength of hybrid ceramic materials, specifically VITA ENAMIC^®^ and Grandio Blocs. The results of the study rejected the null hypothesis, as the bleaching agents significantly affected the color change of hybrid ceramics, with VITA ENAMIC^®^ showing a greater whitening effect compared to Grandio Blocs. Moreover, both the hybrid ceramic material and the bleaching protocol significantly influenced the biaxial flexural strength. Regardless of the bleaching protocol used, Grandio Blocs exhibited significantly higher biaxial flexural strength values than VITA ENAMIC^®^ (*p* < 0.001). Both concentrations of carbamide peroxide (10% and 35%) caused a significant decrease in the biaxial flexural strength of both materials, with VITA ENAMIC^®^ showing a greater reduction compared to Grandio Blocs. These findings highlight the aesthetic impact of bleaching agents on hybrid ceramics, which is particularly important in clinical applications where both the appearance and mechanical properties of restorations are critical.

Subjective approaches (e.g., shade guide) and objective methods (e.g., colorimeter, spectrophotometer, digital image analysis) for evaluating the optical properties of aesthetic restorative materials in dentistry have been extensively studied. Spectrophotometry, for example, has been shown to improve measurement accuracy by 33% [21, 24]. In this study, a spectrophotometer was used to assess color change. The CIE Lab formula, known for its simplicity and wide application, is a commonly used color space that provides a straightforward method for measuring color variations. However, the CIEDE2000 formula, an updated version of CIE Lab, offers greater accuracy, especially for small shifts, and aligns more closely with human visual perception [24].

For NBS (National Bureau of Standards) unit color evaluations, color changes are categorized as follows: changes between 0.5 and 1.5 are considered slight, those between 1.5 and 3 are noticeable, and changes between 3 and 6 are considered appreciable [35]. Understanding the point at which a color difference impacts dental aesthetics (the acceptability threshold) and the level at which it is visually detectable (the perceptibility threshold) is critical. Without this understanding, simply measuring the color difference has limited clinical value. More than half of the studies use a perceptibility threshold of ΔE* = 1, while approximately one-third use ΔE* = 3.7 as the point at which 50% of observers accept the color difference [36]. In this study, the ΔE values of all groups were less than 3.7, indicating that the color changes were within a clinically acceptable range.

The color change (ΔE) in Grandio Blocs showed noticeable changes, while VITA ENAMIC^®^ exhibited an appreciable color change. Specifically, VITA ENAMIC^®^ demonstrated a more pronounced whitening effect compared to Grandio Blocs, which showed a more subtle lightening. This suggests that VITA ENAMIC^®^ was more affected by the bleaching agents, becoming noticeably whiter, while Grandio Blocs underwent a less significant color change. The difference between the two materials may be due to variations in their structures (e.g., polymer chain tightness and crystal homogeneity), which influence the depth of diffusion of bleaching agents [37]. VITA ENAMIC^®^ is a polymer-infiltrated ceramic, composed of a ceramic component with an infiltrated polymer (resin) network made of TEGDMA (Triethyleneglycol dimethacrylate), which makes it more susceptible to the effects of bleaching protocols [38]. TEGDMA increases the hydrophilicity of the composite, leading to a greater susceptibility to color change [39]. In contrast, Grandio Blocs have exceptionally low water absorption, contributing to superior overall performance and longevity [40].

This finding is consistent with Nikanjam et al. (2021), who reported that VITA ENAMIC^®^ showed the most significant color change and whitening, which was deemed clinically unsatisfactory [37]. However, these results differ from those of Ghanem et al. (2017), who stated that the color change was not perceptible and would not necessitate the replacement of the restoration [41]. This discrepancy may be attributed to differences in the application time and concentration of the bleaching agents, as the former study used an in-office bleaching system, whereas this study utilized a home bleaching system. The in-office treatment involved only three applications, each lasting 15 min, requiring less time and fewer applications, which could explain the differing results. Additionally, this study does not align with the findings of Seyidaliyeva et al. (2020), who reported the highest color change in Grandio Blocs exposed to wine.

In this study, the highest color change (ΔE) was observed with carbamide peroxide at 35%, followed by carbamide peroxide at 10%, although the difference was not statistically significant. These results are in agreement with those of Ruwaida Z. Alshali et al. (2020) [21]. However, this finding contradicts the results of Nikanjam et al. (2021) and Karakaya et al. (2020), who reported a more significant color change when hybrid ceramic materials were exposed to a home bleaching agent with a lower concentration and longer exposure time, compared to an in-office bleaching technique with a higher concentration and shorter exposure time [37, 42].

As flexural strength represents the maximum stress before fracture, the biaxial flexural test was selected for this investigation. It indicates the level of tension required to cause the substance to deform before the proportional limit is reached [43]. The null hypothesis was rejected, as the results of the biaxial flexural strength showed that there was a significant effect of the hybrid ceramic material and bleaching protocol on the biaxial flexural strength. Regardless of the bleaching protocol used, Grandio Blocs showed significantly higher values in flexural strength than VITA ENAMIC^®^ (*p* < 0.001). Grandio Blocs have a higher filler content compared to other composite-based blocks. This is achieved by VOCO’s patented nanotechnology, which ensures superior strength and stability [40]. Superior mechanical properties for resin composite blocks (Grandio Blocs) were obtained by composite and curing processing technologies. Resin composite blocks with higher mechanical properties are good options for the fabrication of CAD/CAM indirect restorations [44]. The resin matrix composition of the CAD/CAM blocks may also play an essential role in their mechanical properties. Urethane dimethacrylate (UDMA), the primary monomer of Grandio Blocs, has greater mechanical characteristics than TEGDMA, the primary monomer of VITA ENAMIC^®^, which may explain the superior mechanical qualities of Grandio Blocs [8]. Besides, Grandio Blocs is a nano-ceramic hybrid material that combines ceramic nanosized particles with ordinary filler particles. The use of varied filler sizes allowed for the loading of larger amounts of strong filler particles, which prevented crack propagation and thus increased the material’s strength [8]. Kim et al. and Rastelli et al. reported an intimate relationship between increasing filler load and enhanced flexural strength [45, 46]. This is in agreement with Tokunaga et al. in 2022, who stated that VITA ENAMIC has a microstructure consisting of a micro-sized feldspar porcelain-based ceramic skeleton infiltrated with acrylic resin, while Grandio Blocs consist of nano-sized filler that resists crack propagation and has high flexural strength [7]. Ikeda et al. in 2019 reported that the resin phase has relatively lower physicochemical stability than the ceramic skeleton, leading to water sorption and dissolution into water. Deterioration of the mechanical properties due to water absorption was observed in PICN [47]. A significant decrease in the flexural strength of VITA ENAMIC^®^ and Grandio Blocs was observed after bleaching with carbamide peroxide 35% and carbamide peroxide 10%; however, there was no statistically significant difference between the flexural strength of specimens bleached with carbamide peroxide 35% and those bleached with carbamide peroxide 10%. This decrease may be attributed to free radicals from the bleaching solution attacking the inorganic filler and resin matrix at the interface, causing the fillers to dissolve from the material surface [18]. The lowest value was found in the carbamide peroxide 35% group; this can be explained by the bleaching agents’ release of highly reactive free radicals, which led to the creation of an acidic environment during the bleaching process and structural alterations in the restorative material [48, 49]. This is in agreement with Carvalho AO et al. (2015), who reported that the unbleached control group had significantly higher flexural strength than the group bleached with 38% hydrogen peroxide [50]. These results are partially aligned with those of Shakibafard et al. (2022), who found a statistically significant decrease in the flexural strength of VITA ENAMIC^®^ after bleaching with Opalescence Xtra Boost 40%. However, the decrease in flexural strength of specimens bleached with Opalescence PF 15% was not significant [51]. This is consistent with Yu et al. (2018), who indicated that bleaching with 40% hydrogen peroxide significantly decreased the flexural strength of composites [52], and with Yu et al. (2010), who showed that bleaching with 10% carbamide peroxide significantly decreased the flexural strength of compomer and glass-ionomer [53]. These variances could potentially be related to the different bleaching agents and hybrid materials used.

The Weibull distribution of the failure probability is an effective instrument for examining the statistical fracture of ceramic materials. Less fluctuation in fracture stress and a higher degree of homogeneity between samples are observed with a larger Weibull modulus [54]. For clinical usage, a larger Weibull modulus with less strength scatter is preferred [55]. The Weibull modulus, which describes the nature, severity, and distribution of the defects and the characteristics of strength, is a normalized parameter representing the stress level at which 63% of the specimens fail [56]. Results showed a significant difference in the distribution of both materials (*p* < 0.001). VITA ENAMIC^®^ exhibited a higher Weibull modulus, indicating greater structural reliability and a more predictable failure pattern. In contrast, Grandio Blocs had a higher characteristic strength, meaning it can withstand higher stresses before failure (i.e., higher durability). This suggests that Grandio Blocs has superior overall strength and is less likely to fail under applied stress compared to VITA ENAMIC^®^. The lower characteristic strength and higher Weibull modulus of VITA ENAMIC^®^ suggest that it may be more susceptible to failure at lower stress levels but potentially more consistent when failure does occur.

In clinical terms, materials used for restorations in the oral cavity must be able to withstand masticatory forces. While single-cycle loading to failure is a great indicator of a restoration-tooth system’s fracture strength, it doesn’t reveal much about how damage starts and spreads in the oral environment. Depending on the tooth, sex, and measurement type, it is undoubtedly more than sufficient to withstand maximum bite forces of 70–900 N [57]. Given its higher characteristic strength, Grandio Blocs may be better suited for areas subjected to higher masticatory forces, such as the posterior region, while VITA ENAMIC^®^’s higher Weibull modulus could make it a more predictable option for aesthetic restorations. The difference in characteristic strength between the two materials suggests that Grandio Blocs may offer superior clinical performance in high-stress areas, whereas VITA ENAMIC^®^ could be more suitable for situations where predictable failure patterns are important. This aligns with Homaei et al. (2016), who stated that VITA ENAMIC^®^ has a higher Weibull modulus, indicating less variation in its strength and making it the most reliable material compared to others. However, it has the lowest strength [58].

Based on the findings of this study, it is recommended that practitioners consider the varying bleaching resistance of different hybrid ceramics when selecting materials for bleaching procedures. Materials like Grandio Blocs, which demonstrated greater resistance to bleaching agents, may be more suitable for patients with a history of frequent or intense bleaching treatments. Additionally, hybrid ceramics should be chosen based on their specific properties and intended use; for example, materials such as VITA ENAMIC^®^, which demonstrate color stability, may be preferred for aesthetic restorations, such as anterior restorations, where maintaining a natural appearance is crucial. On the other hand, materials like Grandio Blocs, with higher resistance to bleaching, may be better suited for posterior restorations, particularly in areas subject to high masticatory forces, such as molars, where durability and resistance to wear are essential.

The current study has limitations that should be addressed in future research, including the need for in vivo studies that consider factors such as saliva, masticatory pressures, and longer exposure to bleaching agents, which may impact the mechanical response of restorative materials both before and after bleaching. Therefore, it is important to use caution when applying results to clinical settings. Further in vivo investigations are needed for more trustworthy results. Also, to determine if time affects changes in flexural strength and color, it may be beneficial to apply bleaching chemicals over a longer period and repeat measurements at different time points. Future research should examine how extended exposure to bleaching chemicals affects the surface characteristics and color of ceramics and if these changes are time dependent. In advance, future research could explore hybrid ceramics with enhanced bleaching resistance through filler particle technology or resin matrix formulations. Researchers should explore bleaching techniques’ impact on material qualities to reduce negative impacts on strength and appearance. This could lead to improved material formulations for durable, aesthetically pleasing restorations.

## Conclusions

Within the limitations of the in vitro design, the present study allows the following conclusions to be made:

1. Hybrid ceramic materials respond differently to bleaching, with VITA ENAMIC^®^ showing more noticeable whitening than Grandio Blocs. Thus, Grandio Blocs are more resistant to bleaching.
2. Bleaching agents affect the biaxial flexural strength of both materials, with Grandio Blocs consistently outperforming VITA ENAMIC^®^ in terms of mechanical strength.
3. For high-aesthetic applications, such as anterior restorations where color stability and natural appearance are critical, VITA ENAMIC^®^ is recommended. In contrast, for high-stress areas, such as posterior restorations under heavy masticatory forces, Grandio Blocs would be the preferred choice.

## Data Availability

The datasets used and analyzed during the current study are available from the corresponding author on reasonable request in a link.

## Abbreviations

CAD/CAM: Computer-Aided Design and Computer-Aided Manufacturing
PICN: Polymers Infiltrated Ceramic Network
NBS: National Bureau of Standards
UDMA: Urethane Dimethacrylate
TEGDMA: Triethyleneglycol Dimethacrylate
RNC: Resin-Nano Ceramic
